# Flare risk after oral glucocorticoid bridging with methotrexate or intra‐articular bridging with triple therapy in early rheumatoid arthritis

**DOI:** 10.1111/joim.70094

**Published:** 2026-04-06

**Authors:** Kristina Lend, Jos W. R. Twisk, Frieda A. Koopman, Anna Rudin, Merete Lund Hetland, Till Uhlig, Dan C. Nordström, Michael T. Nurmohamed, Bjorn Gudbjornsson, Kim Hørslev‐Petersen, Marte Schrumpf Heiberg, Tuulikki Sokka‐Isler, Gerdur Grondal, Espen A. Haavardsholm, Mikkel Østergaard, Jon Lampa, Ronald van Vollenhoven

**Affiliations:** ^1^ Division of Rheumatology Department of Medicine Center for Molecular Medicine (CMM) Karolinska Institute Stockholm Sweden; ^2^ Department of Rheumatology and Clinical Immunology Amsterdam University Medical Centers Amsterdam the Netherlands; ^3^ Department of Epidemiology and Data Science Amsterdam University Medical Centres Amsterdam the Netherlands; ^4^ Department of Rheumatology Sahlgrenska University Hospital Gothenburg Sweden; ^5^ Department of Rheumatology and Inflammation Research Institute of Medicine, Sahlgrenska Academy Gothenburg Sweden; ^6^ Department of Clinical Medicine Faculty of Health and Medical Sciences University of Copenhagen Copenhagen Denmark; ^7^ Copenhagen Center for Arthritis Research Center for Rheumatology and Spine Diseases Rigshospitalet Glostrup Glostrup Denmark; ^8^ Center for Treatment of Rheumatic and Musculoskeletal Diseases (REMEDY) Diakonhjemmet Hospital Oslo Norway; ^9^ Faculty of Medicine University of Oslo Oslo Norway; ^10^ Departments of Medicine and Rheumatology Helsinki University Hospital Helsinki Finland; ^11^ Department of Internal Medicine University of Helsinki Helsinki Finland; ^12^ Amsterdam Rheumatology and Immunology Center, Reade Amsterdam the Netherlands; ^13^ Centre for Rheumatology Research Landspitali University Hospital Reykjavik Iceland; ^14^ Faculty of Medicine University of Iceland Reykjavik Iceland; ^15^ Danish Hospital for Rheumatic Diseases University Hospital of Southern Denmark Sønderborg Denmark; ^16^ Department of Regional Health Research University of Southern Denmark Odense Syddanmark Denmark; ^17^ Faculty of Health Sciences University of Eastern Finland Kuopio Finland; ^18^ Rheumatology, Hospital Nova of Wellbeing Services County of Central Finland Jyväskylä Finland; ^19^ Department of Gastroenterology Dermatology and Rheumatology Karolinska University Hospital Stockholm Sweden

**Keywords:** flare, intra‐articular glucocorticoid injections, oral glucocorticoids, rheumatoid arthritis, tapering and discontinuation

## Abstract

**Objectives:**

To investigate, in early rheumatoid arthritis, whether bridging with glucocorticoids (GCs) is associated with an increased risk of flare following GC tapering and withdrawal.

**Methods:**

A total of 810 NOrdic Rheumatic Diseases Strategy Trials And Registries (NORD‐STAR) patients were included in this post hoc analysis: all received methotrexate (MTX), in addition to which 135 patients received oral GC bridging therapy (‘oral GC group’); 80 received intra‐articular (IA) GC bridging therapy, sulfasalazine and hydroxychloroquine (‘injection GC group’); and 595 received one of three (certolizumab pegol, abatacept and tocilizumab) biologic disease‐modifying antirheumatic drugs (bDMARDs), hereafter the (‘bDMARD group’). Clinical disease activity index (CDAI) flares (≥4.5 increase in CDAI score) were assessed longitudinally.

**Results:**

Up to 48 weeks, flare occurred at least once in 43% of oral GC, 24% of injection GC and 28% of bDMARD patients. Over‐time relative risk (RR) was higher with oral GC bridging (adjusted RR, 1.54; 95% CI, 1.16–2.03) but similar with injection GC bridging (adjusted RR 0.93; 95% CI, 0.54–1.55) versus bDMARD. Flare rates were numerically higher in the oral GC versus bDMARD group at all time points (12, 24, 32, 40 and 48 weeks), with a significant difference at w.40, the visit after protocol‐defined GC discontinuation; at this visit 27% of patients who discontinued GC experienced flare; 29% among those in remission; 33% remained on low‐dose prednisolone at 48 weeks.

**Conclusion:**

Discontinuation of oral GC bridging therapy on background MTX is associated with increased flare risk, even among patients in remission. Flare rates with IA GC bridging plus triple therapy did not differ from the bDMARD group.

**Trial registration number:**

EudraCT 2011‐004720‐35; ClinicalTrials.gov NCT01491815.

## Graphical abstract

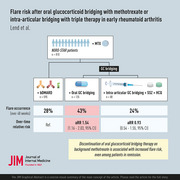

**Abbreviations**
ABA: abatacept
ACPA: anti‐citrullinated protein antibody
bDMARD: biologic disease‐modifying antirheumatic drug
CDAI: clinical disease activity index
CZP: certolizumab pegol
DAS28‐CRP: disease activity score of 28 joints, based on C‐reactive protein
GC: glucocorticoid
GEE: generalised estimating equations
HCQ: hydroxychloroquine
IA: intra‐articular
MTX: methotrexate
NORD‐STAR: NOrdic Rheumatic Diseases Strategy Trials And Registries
RA: rheumatoid arthritis
SSZ: sulfasalazine
TCZ: tocilizumab

## Introduction

Glucocorticoids (GCs) are often part of the initial treatment of early rheumatoid arthritis (RA), serving as bridging therapy to achieve rapid suppression of inflammation and symptoms until slow‐acting conventional synthetic disease‐modifying antirheumatic drugs (csDMARDs), such as methotrexate (MTX), become effective [1, 2]. GCs are highly effective antirheumatic drugs, but their long‐term use has been associated in a dose‐ and duration‐dependent manner with various adverse events, such as osteoporosis [3], exacerbation of diabetes mellitus [4] and cardiovascular disease [5, 6]. When used as short‐term bridging therapy, tapering of GC may unmask residual disease activity, thus inducing a flare.

Previous reports have shown that approximately 40% of early RA patients experienced a flare after the initial discontinuation of oral GCs [7]. However, the proportion of flares attributable to oral GC discontinuation versus those occurring randomly remains unclear, as does the frequency of flares during the tapering phase of the oral GC bridging strategy. Data on flare rates associated with intra‐articular (IA) GC injection bridging are scarce [8], highlighting the need for further investigation.

Previous research has employed varying criteria for the definition of flare. Some studies have defined flare as the failure to maintain treatment target [7], whereas others have defined it based on one of the following: a switch or increase in DMARD dose; GC reinitiation; a DAS28 increase of more than 0.6 units [9, 10]; a rheumatologist's judgement [10]; or a patient‐perceived flare [11].

Clinical disease activity index (CDAI) flare, defined by an increase of 4.5 units, is a newly validated flare definition that accounts for absolute changes in disease activity, enabling objective and consistent measurement of the outcome [11].

The investigator‐initiated NOrdic Rheumatic Diseases Strategy Trials and Registries (NORD‐STAR) randomised controlled trial included DMARD‐naïve RA patients who were randomised to receive conventional treatment or one of the three biologic treatments (certolizumab pegol [CZP], abatacept [ABA] or tocilizumab [TCZ]). All patients received MTX. In the active conventional therapy arm, GC bridging involved two country‐based non‐randomised strategies: oral GC bridging therapy, applied for all patients in Sweden, Norway, the Netherlands and Iceland; and IA GC injection bridging therapy, with two additional csDMARDs (sulfasalazine [SSZ] and hydroxychloroquine [HCQ]), applied for all patients in Denmark and Finland [12, 13, 14].

In this post hoc analysis, we aimed to analyse CDAI‐increase‐defined flare rates over a 48‐week period with two GC bridging strategies—oral GC bridging plus MTX and IA GC injection bridging plus triple therapy—with biologic disease‐modifying antirheumatic drug (bDMARD) plus MTX as the reference regimen.

## Methods

### Study design and participants

In the NORD‐STAR trial, 812 patients with early RA (according to the 2010 American College of Rheumatology (ACR)/European Alliance of Associations for Rheumatology (EULAR) classification criteria), aged 18 or older, naive to DMARDs, with symptom duration less than 24 months, moderate to severe disease activity score of 28 joints (DAS28‐CRP >3.2), and with anti‐citrullinated protein antibody (ACPA), rheumatoid factor (RF) positivity or increased CRP (≥10 mg/L), were enrolled [12, 13, 14]. Informed consent was obtained from all patients who participated in the trial.

### Randomisation

In the NORD‐STAR trial, participants were randomly assigned in a 1:1:1:1 ratio stratified by country, sex and ACPA status into one of the following treatment arms:

- Treatment arm 1 received active conventional treatment, comprising two non‐randomised regimes applied according to patients’ country of residence.
  − 1A (‘oral GC group’) (Sweden, Norway, the Netherlands and Iceland): MTX plus oral prednisolone, tapered from 20 to 5 mg per day within 9 weeks and with protocol‐described discontinuation by Week 36.
  − 1B (‘injection GC group’) (Denmark and Finland): MTX plus SSZ (2 g per day), plus HCQ (35 mg/kg per week or 200 mg per day) and mandatory IA GCs (triamcinolone hexacetonide or equivalent) injected into swollen joints (up to four joints and maximally 80 mg per visit).
- Treatment arm 2 received MTX plus CZP (200 mg subcutaneously administered every other week, with a loading dose of 400 mg at Weeks 0, 2 and 4).
- Treatment arm 3 received MTX plus ABA (125 mg subcutaneously administered every week).
- Treatment arm 4 received MTX plus TCZ (8 mg/kg intravenously administered every 4 weeks or 162 mg subcutaneously administered every week) [12].

For the current analysis, the pooled bDMARD arms 2–4 (here termed the ‘bDMARD group’) served as the reference for the oral GC group and for the injection GC group.

Seventeen Finnish patients initially randomised to TCZ but reallocated to receive active conventional therapy due to unavailability of TCZ were included in the injection GC group.

Oral GCs were permitted only in patients assigned to the oral GC group. In the injection GC group, IA GC injections were given whenever a swollen joint was detected during a visit. In the bDMARD group, IA GC injections were allowed on demand up to Week 12, after which a maximum of 40 mg (triamcinolone hexacetonide or equivalent) could be administered every 12 weeks, and in the oral GC group, they were provided when clinically necessary. In each treatment group, the use of IA GC injections was prohibited during Weeks 20–24 and 44–48 [12, 13, 14].

Concomitant MTX was initiated on Day 1 (10–15 mg orally) in all treatment groups, with a step‐up regimen aiming to achieve the target weekly dose of 25 mg by Week 4. Investigators could adjust the dosing or administration route of MTX as clinically necessary if the 25 mg dose was not tolerated. Patients received the highest tolerated MTX dose [12, 15].

### Outcomes

This substudy had three co‐primary outcomes: (i) the proportion of patients experiencing CDAI flare at least once within the first 48 weeks of treatment, (ii) the proportion of patients experiencing CDAI flare at the visit following oral GC discontinuation, and (iii) the risk of CDAI flare with two different GC bridging strategies—oral GC bridging plus MTX and IA GC injection bridging plus triple therapy—compared with bDMARD plus MTX as the reference regimen. CDAI flare was defined as an increase of at least 4.5 units in CDAI score [11] since the previous visit.

### Statistical analysis

CDAI flare was analysed longitudinally at five time points (12, 24, 32, 40 and 48 weeks) with logistic generalised estimating equation (GEE) analysis. At Week 12, flare was defined as an increase in the CDAI score of at least 4.5 units from Week 4. This definition was consistently applied at subsequent intervals with changes calculated relative to the prior visit (e.g., Week 24 relative to Week 12 and Week 32 relative to Week 24). Two main analyses were performed. One GEE analysis with the treatment group variable (i.e., two dummy variables) as independent variable in order to estimate the difference on average over time. In the second analysis, time (treated as a categorical variable and represented by dummy variables) and the interaction between the treatment group and time were added to model in order to estimate the difference between the treatment groups at the different time points. GEE analysis was performed with a logit link and an exchangeable correlation matrix.

Although the protocol specified oral GC discontinuation was at Week 36, deviations occurred: 33% of patients were unable to discontinue oral GCs by Week 48. Discontinuing occurred also outside the intended window (the target was Week 36). Subgroup analyses were therefore conducted, including oral GC patients who discontinued GC bridging between Weeks 32 and 40, as well as all patients from injection GC group and bDMARD group with available flare assessments at Week 40.

The second subgroup analysis was conducted to assess whether being in remission prior to discontinuation of oral GCs influenced the outcome. This subgroup analysis included patients from the previous subgroup, with the additional requirement of CDAI remission at Week 32, prior to the Week 40 flare assessment. Flare at the visit following oral GC treatment discontinuation (at Week 40) was estimated with logistic regression.

Relative risks (RRs) were derived from odds ratios. Analyses were performed crudely and adjusted for sex, ACPA status, country, age, BMI, DAS28‐CRP and smoking status at baseline.

Pearson's chi‐square tests were used to compare observed categorical frequencies of outcomes between the treatment groups.

Statistical analyses were performed in Stata (version 18) and SPSS (version 28). GraphPad Prism 10 was used for the figures and graphical abstract with BioRender.

The NORD‐STAR trial is registered with EudraCT (2011‐004720‐35) and ClinicalTrials.gov (NCT01491815).

## Results

**Fig. 1:**
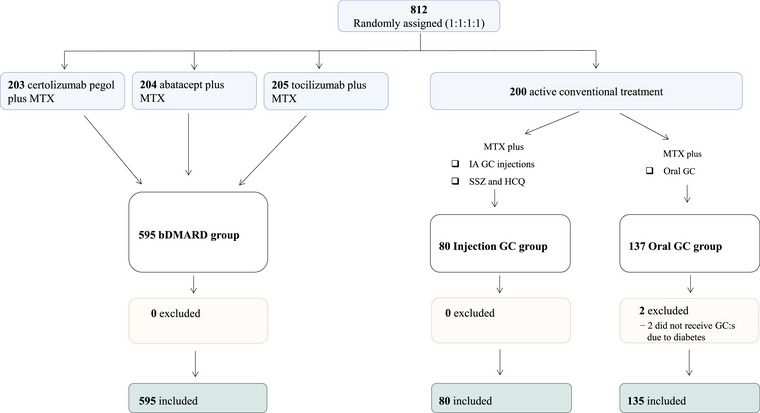
Flowchart of patients included in the analysis. Seventeen Finnish patients randomised to tocilizumab received ‘injection GC group’ treatment due to tocilizumab unavailability and were analysed as part of the ‘injection GC group’. bDMARD, biologic disease‐modifying antirheumatic drug; GC, glucocorticoid; HCQ, hydroxychloroquine; IA, intra‐articular; MTX, methotrexate; SSZ, sulfasalazine.

A total of 812 patients underwent randomisation in the NORD‐STAR trial [13]. Two patients were excluded from this post‐hoc analysis for not receiving the assigned oral GCs because of diabetes, leaving 810 patients included in this post‐hoc analysis: 135 in the oral GC group, 80 in the injection GC group and 595 in the bDMARD group (Fig. 1).

At baseline, the injection GC group had the longest time since diagnosis and the lowest tender joint count. Otherwise, the baseline characteristics were similar across groups and are shown in Table 1. Early terminations occurred before Week 48 in 29/135 (22%) patients in the oral GC group, 9/80 (11%) in the injection GC group and 90/595 (15%) in the bDMARD group (*p* = 0.094).

**Table 1:** Baseline characteristics and medications of interest in patients with early rheumatoid arthritis, stratified by treatment group.

Baseline characteristics	bDMARD group	Injection GC group	Oral GC group
*n* (patients)	595	80	135
Treatment added to MTX	bDMARD	IA GC injections, SSZ and HCQ	Oral GC
Female (%)	69	73	70
Age, years	54.2 (14.8)	54.0 (14.5)	54.3 (15.0)
Diagnosis duration, days, median (IQR)	7 (1–18)	12 (0–26)	6 (0–9)
Body‐mass index, kg/m^2^	26.1 (5.0)	26.6 (5.6)	26.8 (5.3)
**Smoking (%)**			
Current smoker	23	19	17
Former smoker	37	41	44
Non‐smoker	40	40	39
ACPA positive (%)	82	85	81
RF positive (%)	75	76	75
CDAI score	27.7 (11.8)	26.0 (11.2)	29.7 (12.3)
DAS28‐CRP^a^	5.0 (1.1)	4.9 (1.0)	5.1 (1.1)
Swollen joint count (28 joints)	7.8 (5.0)	7.0 (4.7)	8.6 (5.3)
Tender joint count (28 joints)	9.1 (5.9)	8.5 (5.7)	10.6 (6.7)
Patient's global assessment of disease activity (mm)	58 (23)	60 (25)	54 (22)
Physician's global assessment of disease activity (mm)	50 (19)	45 (19)	50 (19)
C‐reactive protein, mg/L, median (IQR)	11 (4–23)	10 (4–23)	13 (4–26)
**Medications of interest**			
Cumulative number of IA GC injections before Week 16, median (IQR)	1 (0–3)	6 (4–9)	0 (0–0)
Cumulative number of IA GC injections between 16 and 48 weeks, median (IQR)	0 (0–0)	0 (0–1)	0 (0–0)
MTX dose at 24 weeks (mg)	20.4 (6.2)	22.1 (4.7)	22.5 (4.0)
MTX dose at 48 weeks (mg)	19.2 (6.8)	22.0 (4.7)	21.9 (4.6)

*Note*: Data are mean (SD), unless otherwise specified. bDMARD refers to a treatment with one of the biologics (certolizumab pegol, abatacept or tocilizumab).

Abbreviations: ACPA, anti‐citrullinated protein antibodies; bDMARD, biologic disease‐modifying antirheumatic drug; CDAI, clinical disease activity index; DAS28‐CRP, disease activity score of 28 joints, based on C‐reactive protein; GC, glucocorticoid; HCQ, hydroxychloroquine; IA, intra‐articular; IQR, interquartile range; MTX, methotrexate; RF, rheumatoid factor; SSZ, sulfasalazine.

^a^ DAS28‐CRP was replaced with DAS28‐ESR for two patients.

The flare assessments could not be done in a subset of patients due to early termination prior to the first flare assessment at Week 12 or missed visits. This occurred in 5/135 (4%) patients in the oral GC group, 5/80 (6%) in the injection GC group and 30/595 (5%) in the bDMARD group.

### Risk of flare over time and at individual time points

Up to 48 weeks, a flare occurred at least once in 56/130 (43%) patients in the oral GC group, 18/75 (24%) in the injection GC group and 160/565 (28%) in the bDMARD group (*p* = 0.002).

**Fig. 2:**
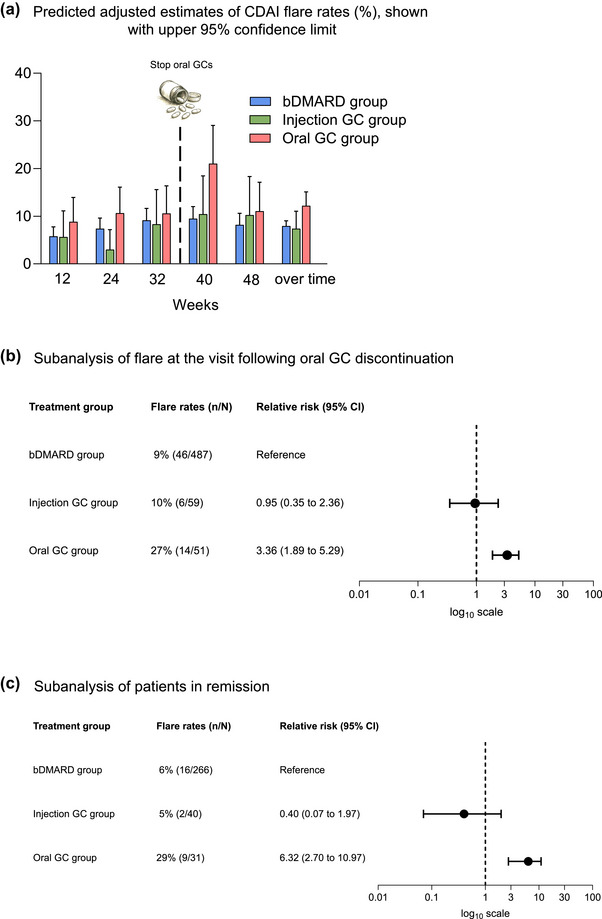
(a) Predicted adjusted estimates of CDAI flare rates, shown with 95% confidence intervals; (b) adjusted relative risk of CDAI flare at the visit following oral GC discontinuation, among patients who discontinued GCs between Weeks 32 and 40, injection GC group compared with the reference bDMARD group. (c) The second subgroup analysis was restricted to patients in remission; bDMARD refers to a treatment with one of the biologics (certolizumab pegol, abatacept or tocilizumab). bDMARD, biologic disease‐modifying antirheumatic drug; CDAI, clinical disease activity index; GC, glucocorticoid.

Table 2 and Fig. 2a show the results of the longitudinal GEE analyses. The risk of a CDAI‐increase‐defined flare over time was higher in the oral GC group (adjusted RR: 1.54 [95% CI: 1.16–2.03]) compared with the reference risk (i.e., the bDMARD group). Analysis at individual time points (12, 24, 32, 40 and 48 weeks) showed that the probability of flare was consistently numerically higher in the oral GC group at all visits, with a significant difference at Week 40 (adjusted RR: 2.25 [95% CI: 1.39–3.44]), the visit following protocol‐described GC discontinuation at 36 weeks, compared with the bDMARD group. In contrast, the flare rates in the injection GC group did not differ from those in the bDMARD group over time (adjusted RR: 0.93 [95% CI: 0.54–1.55]) or at any individual time points, despite the majority of IA injections being administered early in the study. The median number of cumulative injections given between Weeks 16 and 48 was 0 (IQR 0–1) (Table 1), with values ranging from 0 to 9 in the injection GC group.

**Table 2:** Risk of CDAI flare up to 48 weeks in the GC‐treated groups compared with the reference bDMARD group.

		Injection GC group		Oral GC group	
Crude	bDMARD group	Relative risk (95% CI)	*p*	Relative risk (95% CI)	*p*
Over time	Reference	0.91 (0.57 –1.42)	0.68	1.57 (1.21–2.03)	0.00
12 weeks	Reference	0.96 (0.34–2.54)	0.94	1.56 (0.80–2.92)	0.19
24 weeks	Reference	0.40 (0.10–1.52)	0.18	1.49 (0.81–2.61)	0.20
32 weeks	Reference	0.89 (0.36–2.07)	0.80	1.19 (0.64–2.10)	0.57
40 weeks	Reference	1.09 (0.48–2.31)	0.83	2.24 (1.41–3.37)	0.00
48 weeks	Reference	1.25 (0.54–2.65)	0.60	1.38 (0.73–2.48)	0.31
**Adjusted**					
Over time	Reference	0.93 (0.54–1.55)	0.78	1.54 (1.16–2.03)	0.00
12 weeks	Reference	0.97 (0.33–2.65)	0.96	1.54 (0.77–2.91)	0.22
24 weeks	Reference	0.40 (0.09–1.62)	0.21	1.45 (0.78–2.57)	0.23
32 weeks	Reference	0.91 (0.35–2.18)	0.84	1.16 (0.61–2.10)	0.65
40 weeks	Reference	1.10 (0.47–2.40)	0.82	2.25 (1.39–3.44)	0.00
48 weeks	Reference	1.25 (0.51–2.80)	0.61	1.36 (0.71–2.47)	0.35

*Note*: bDMARD refers to a treatment with one of the biologics (certolizumab pegol, abatacept or tocilizumab). Nominal *p*‐values; no adjustment for multiple comparisons.

Abbreviations: bDMARD, biologic disease‐modifying antirheumatic drug; GC, glucocorticoid; HCQ, hydroxychloroquine; IA, intra‐articular; MTX, methotrexate; SSZ, sulfasalazine.

### Subgroup analysis of flare at the visit following oral GC discontinuation

Of the patients in the oral GC group who remained in the trial at Week 48, 35/106 (33%) were still receiving oral GCs at 48 weeks, and 20/106 (19%) discontinued oral GCs outside the intended window (Week 36 was target). The remaining oral GC patients, 51/106 (48%), who discontinued oral GC between Weeks 32 and 40 were included in the subgroup analysis, as well as 59/80 (74%) patients from the injection GC group and 487/595 (82%) from the bDMARD group who had available flare assessment at Week 40.

Flare occurred at the visit following oral GC discontinuation (Week 40) in 14/51 (27%) in the oral GC group, 6/59 (10%) in the injection GC group and 46/487 (9%) in the bDMARD group (*p* < 0.001).

The RR of flare was higher in the oral GC group (adjusted RR: 3.36 [95% CI: 1.89–5.29]) but did not differ in the injection GC group patients (adjusted RR: 0.95 [95% CI: 0.35–2.36]) when compared with the bDMARD group (Fig. 2b).

### Subgroup analysis of flare among patients in remission

To explore whether the risk of flare could be reduced by discontinuing oral GCs at the time of remission, we performed additional subgroup analysis of patients in CDAI remission. A total of 337 patients were in CDAI remission at Week 32 and were included in this subgroup analysis: 31 from the oral GC group who discontinued GCs between Week 32 and 40, 40 from the injection GC group, and 266 from the bDMARD group. At the visit following oral GC discontinuation (Week 40), a flare had occurred in 29% (9/31) of oral GC group, 5% (2/40) of the injection GC group and 6% (16/266) of the bDMARD group patients.

The results were in line with those of the main and overall subgroup analyses. RR of flare was higher in the oral GC group after discontinuation of oral GCs (adjusted RR: 6.32 [95% CI: 2.70–10.97]), compared with the bDMARD group, whereas flare rates in the injection GC group (adjusted RR: 0.40 [95% CI: 0.07–1.97]) did not differ from the bDMARD group (Fig. 2c).

Unadjusted subgroup analyses results were similar and are shown in Fig. S1.

### Disease activity before and after flare

To understand the pattern of disease activity following flares, we first examined disease activity in the subgroup analyses, stratified by flare occurrence at Week 40. In patients who flared, disease activity appeared to be generally higher even at Week 48 and had not returned to pre‐flare level regardless of treatment (Figs. S2 and S3).

To explore the duration of increased disease activity following an early flare, we stratified patients by the first flare assessment status at Week 12 (worsening of disease from Week 4) and assessed disease activity until 48 weeks. Disease activity at Week 4 was comparable between patients who did and did not flare at Week 12; however, disease activity appeared to remain higher through Week 48 in those who flared in oral GC group and bDMARD group. The interpretation for injection GC group is limited by the small sample size (Fig. S4).

## Discussion

In this post hoc analysis of early RA, we compared CDAI‐increase‐defined flare rates up to 48 weeks in two GC bridging strategies: longer term oral GC bridging strategy (oral GCs plus MTX), and IA GC injection bridging strategy (IA GC injections plus triple therapy (MTX/SSZ/HCQ)), with the pooled bDMARD group (CZP, ABA or TCZ, all in combination with MTX) as the reference regimen.

During the first 48 weeks, flares were most common in the oral GC group, with at least one flare experienced in 43% of patients. Flare rates in injection GC group (24%) were similar to the reference bDMARD group (28%).

To our knowledge, no other studies have analysed CDAI‐increase‐defined flare rates longitudinally or compared flare rates in two different GC bridging strategies (oral administration with systemic effect vs. IA GC injection with local effect), with bDMARD treatment as the reference.

Flare rates were assessments at five time points (from Week 12 up to Week 48) to capture its patterns.

The flare rates were consistently numerically higher in the oral GC group compared with the bDMARD group, with the largest difference at the visit following protocolised GC discontinuation. When GCs were administered as IA injections on background triple therapy, flare rates did not differ from bDMARD group, neither over time nor at any time point.

One potential influencing factor may be concomitant csDMARDs. Thus, patients in the oral GC group received MTX, whereas patients in the injection GC group received triple therapy. Some studies have reported clinical benefit from combining MTX with other csDMARDs compared with single‐agent csDMARD therapy [16, 17], whereas others have not [18, 19]. In the NORD‐STAR trial, oral GC group had numerically higher remission rates until Week 24, but short thereafter, the curves crossed in the favour of the injection GC group [20]. However, flare rates were consistently highest in the oral GC group.

The differences in flare rates, and whether they reflect the administration route of GC (IA injection vs. oral) or the background use of csDMARD(s), remain difficult to determine with certainty in combination therapies. However, pharmacokinetic and pharmacodynamic considerations provide some guidance. csDMARDS are slow‐acting and their effects diminish gradually; moreover, during the first 48 weeks of our study, no protocolised discontinuation was required for csDMARD(s), restricting discontinuation only to GCs.

In the injection GC group, the majority of injections were administered early in the trial. Notably, there were no higher flare rates in the injection GC group compared with bDMARD neither over time nor at any time point. It is possible that this may reflect a prolonged effect of IA GC injections, especially when administered for the first time, with nearly two‐thirds of these joints remaining relapse‐free after 1 year [8]. We cannot exclude the possibility of continuous effects of triple therapy but this seems less likely as the single explanation for lower flare rates in this group.

The higher flare rate observed in the oral GC group highlights the contribution of oral GCs—through systemic exposure—to disease control beyond MTX, which is particularly crucial in a subset of patients for whom MTX alone does not provide full disease control.

To explore whether the risk of flare could be reduced by discontinuing oral GCs at the time of remission, we performed a subgroup analysis. In the oral GC group, remission at time of discontinuation did not appear protective; flare rates were similar between the remission oral GC subgroup (29%) and the overall oral GC group who discontinued GCs before Week 40 (27%). In contrast, remission seemed to offer some protection in the injection GC group and in the bDMARD group, where flare rates were slightly lower (5% in the injection GC group and 6%, in the bDMARD group) than in the overall group (10% and 9%, respectively).

Direct comparison with other trials involving protocolised long‐term low‐dose oral GC discontinuation is limited due to differences in study designs, treatment targets and outcome measures. Our oral GC bridging therapy group results are best compared with the BeSt study [21] and IMPROVED study [22] with regard to the comparable protocol‐specified duration of GC treatment and early RA population. Flare rates following GC discontinuation were higher in both the BeSt (40%) and the IMPROVED (39%) studies [7], compared with our study (27% among the overall GC discontinuation group; 29% among those in remission). These differences may be attributed to variations in the definition of flare. In the BeSt study, oral GCs were discontinued when DAS ≤2.4, whereas in the IMPROVED study, discontinuation occurred when DAS was <1.6. Discontinuation was considered successful if the treat‐to‐target goal was maintained at the next visit [7]. However, these definitions required only minimal increase in disease activity in patients near the cut‐off threshold to be classified as experiencing a flare. Moreover, both studies focused on patients who met treat‐to‐target criteria for GC discontinuation, excluding those patients who never achieved the treatment targets in the first place. In contrast, we used the CDAI‐increase‐defined definition of flare, based on absolute changes in the CDAI score [11], including all patients for the duration of trial participation.

Oral GC bridging therapy is intended for short‐term use to manage symptoms until the full therapeutic effects of csDMARD are achieved [1, 2]. However, in our study, 33% of the oral GC group, patients who completed the 48‐week period, were still on low‐dose GCs at Week 48. Inability to taper GCs may signal the need for treatment intensification or a need for personalised tapering schedules. Deviations from the protocolised GC discontinuation have also been observed in other trials, with 29% reported in the BeSt study and 34% in the IMPROVED study, where GC discontinuation was either never attempted or could not be studied [7]. In a study combining individual patient data from trial‐based cohorts, most patients on protocolised GC bridging schedules were able to discontinue treatment within 2 years [2]. In real‐world practice, nearly half of patients with established RA were prescribed GCs, with most maintaining a low‐dose regimen for 2 years, highlighting potential challenges in discontinuing the treatment [23]. In a real‐world ‘treat‐to‐target’ cohort with a median symptom duration of 24 months (IQR 8.5–96.0), the cumulative probabilities of GC discontinuation were 27%, 48% and 59% at Years 1, 2, and 3, respectively [10], which are far from the recommended guidelines [1]. The potential benefits of long‐term GC use, particularly at lower doses, or harms, remain a matter of debate. This uncertainty is further complicated, as both higher inflammatory activity and cumulative GC doses both contribute to cardiovascular disease. In this context, shortening or extending the duration of oral bridging therapy—guided by individual patient needs—may be a reasonable consideration.

Flares have a considerable impact on patients’ wellbeing, extending beyond joint pain and swelling [24]. Therefore, we first explored whether disease activity would return to pre‐flare levels in the subgroup analysis, stratified by flare occurrence at Week 40. Second, we assessed the duration of flare impact by stratifying patients who flared or did not flare at the first flare assessment time point (at Week 12) and examined the disease activity up to Week 48. In general, patients in the oral GC group and bDMARD group who experienced a flare appeared to have higher disease activity at the subsequent visit(s) compared with those who did not. Interpretation for injection GC group is limited by the small sample size. Our findings raise the possibility that an early flare may influence subsequent disease activity not only when a medication is tapered or discontinued but also in patients receiving full‐dose bDMARD with maximally tolerated MTX dose.

We acknowledge several limitations in this study, including some missing data (Table S3), and variation in the timing of GC discontinuation, which affected the GC‐free period prior to the flare assessment. However, the regular visit intervals helped minimise this effect. Furthermore, in the injection GC group, GC injections could be administered into swollen joints at any visit, except 20–24 and 44–48 weeks. Although we cannot exclude the possibility that the injection GC group treatment strategy provided some protection against flares, most swollen joints resolved early. The cumulative number of IA injections between Weeks 16 and 48 was low, with a median of 0 (IQR 0–1), with values ranging from 0 to 9. To put this estimation into context, one swollen joint in the 28‐joint count added one point to the CDAI score, whereas 4.5 points were required for CDAI flare. Although active conventional treatment was randomised, the specific regimen (either oral GCs plus MTX or IA GCs injections plus triple therapy) was determined by the patients’ country of residence. IA GC injections were administered with three csDMARDs (MTX/SSZ/HCQ), whereas oral GCs were given with one csDMARD (MTX), which may have contributed to differences in flare rates. *p* values are nominal and were not adjusted for multiple comparisons and therefore should be interpreted with caution.

A strength of our study is the use of a standardised definition of CDAI flare, applied consistently irrespective of prior disease activity. Patients were scheduled for regular study visits, allowing consistent assessment of outcomes across all groups. Nonetheless, tight assessments may not capture flares that develop gradually over several consecutive visits.

In conclusion, our findings suggest that discontinuation of oral GC bridging therapy on background MTX is associated with a flare risk that exceeds the reference risk (bDMARD plus MTX), even in patients in remission at the time of discontinuation. Flare rates did not differ from the reference risk when GCs were administered intra‐articularly into swollen joints as a bridging strategy in combination with triple therapy (MTX/SSZ/HCQ). When choosing longer term, low‐dose GC bridging strategy for early RA management, the increased risk of a flare upon discontinuation should be considered.

## Author contributions

Kristina Lend contributed to the study conception and design, performed the analyses, contributed to data interpretation and wrote the first draft of the manuscript. Jos W. R. Twisk, Merete Lund Hetland, Frieda A. Koopman, Jon Lampa and Ronald van Vollenhoven contributed to the design or the interpretation of data. Kristina Lend, Anna Rudin, Merete Lund Hetland, Till Uhlig, Dan C. Nordström, Michael T. Nurmohamed, Bjorn Gudbjornsson, Kim Hørslev‐Petersen, Marte Schrumpf Heiberg, Tuulikki Sokka‐Isler, Espen A. Haavardsholm, Gerdur Grondal, Mikkel Østergaard, Jon Lampa and Ronald van Vollenhoven contributed to the acquisition of data. Merete Lund Hetland, Till Uhlig, Dan C. Nordström, Bjorn Gudbjornsson, Kim Hørslev‐Petersen, Gerdur Grondal, Espen A. Haavardsholm, Mikkel Østergaard and Ronald van Vollenhoven designed the original NORD‐STAR protocol. All authors critically read, reviewed and approved the final manuscript. Kristina Lend is guarantor of the overall content, accepts full responsibility for the work and the conduct of the study, has access to the data, and controlled the decision to publish.

## Conflict of interest statement

Merete Lund Hetland reports institutional grants from AbbVie, Bristol Myers Squibb, Eli Lilly, MSD, Pfizer, Sandoz, Novartis, Nordforsk and UCB; speaker honoraria from Medac, Novartis, Pfizer, Sandoz and UCB; institutional data safety monitoring board or advisory board fees from AbbVie. Merete Lund Hetland has chaired the steering committee of the Danish Rheumatology Quality Registry (DANBIO, DRQ), which receives public funding from the hospital owners and funding from pharmaceutical companies. Merete Lund Hetland co‐chairs EuroSpA, which generates real‐world evidence of treatment of psoriatic arthritis and axial spondyloarthritis based on secondary data and is partly funded by Novartis and UCB. Dan C. Nordström reports research grant from MSD; consulting fees from Bristol Myers Squibb, Lilly, Novartis, Pfizer and UCB; speaker honoraria from Pfizer and UCB; participation on a data safety monitoring board or advisory board fees from UCB. Mikkel Østergaard reports institutional grants from AbbVie, Amgen, Bristol Myers Squibb, Merck, Celgene, Eli Lilly, Novartis and UCB; personal speaker honoraria from AbbVie, Bristol Myers Squibb, Boehringer‐Ingelheim, Celgene, Eli Lilly, Galapagos, Gilead, Hospira, Janssen, MEDAC, Merck, Novartis, Novo, Orion, Pfizer, Regeneron, Roche, Sandoz, Sanofi and UCB; participation on a data safety monitoring board or advisory board personal fees from AbbVie, Bristol Myers Squibb, Boehringer‐Ingelheim, Celgene, Eli Lilly, Galapagos, Gilead, Hospira, Janssen, MEDAC, Merck, Novartis, Novo, Orion, Pfizer, Regeneron, Roche, Sandoz, Sanofi and UCB. Espen A. Haavardsholm reports institutional grant from research council of Norway; personal speaker honoraria from Pfizer, UCB and Novartis; and participation on a data safety monitoring board or advisory board fees from AbbVie, Pfizer and Eli Lilly. Till Uhlig reports personal speaker honoraria from AbbVie, Eli Lilly, Pfizer, UCB and Novartis. Ronald van Vollenhoven reports institutional support for the present manuscript from Bristol Myers Squibb; Institutional grants for research or education from Alfasigma, AstraZeneca, Bristol Myers Squibb, Galapagos, MSD, Novartis, Pfizer, Roche, Sanofi and UCB; consulting fees from AbbVie, AstraZeneca, Biogen, Bristol Myers Squibb, Galapagos, GSK, Janssen, Pfizer, RemeGen and UCB; speaker honoraria from AbbVie, AstraZeneca, Bristol Myers Squibb, Galapagos, GSK, Janssen, Pfizer and UCB; and participation on a data safety monitoring board or advisory board fees from AbbVie, AstraZeneca, Biogen, Bristol Myers Squibb, Galapagos, GSK, Janssen, Pfizer, RemeGen and UCB. All other authors declare no competing interests.

## Supporting information




**Table S1**: Baseline characteristics and medications of patients included in the subgroup analysis (*n* = 597).
**Table S2**: Baseline characteristics and medications of patients included in the remission subgroup analysis (*n* = 337).
**Table S3**: Missingness of CDAI and CDAI flare data, stratified by treatment group.
**Figure S1**: Crude subgroup analyses.
**Figure S2**: Disease activity stratified by flare status at Week 40.
**Figure S3**: Disease activity stratified by flare status at Week 40 for the remission subgroup.
**Figure S4**: Assessment of the influence of Week 12 CDAI flare on disease activity up to Week 48.

## Data Availability

NORD‐STAR data will not be shared publicly. Access to the NORD‐STAR data is organized according to a strict data access procedure. For all types of access, a research proposal must be submitted for evaluation by the NORD‐STAR steering committee. The evaluation is performed to align the goals of the researchers with the goals of NORD‐STAR. Further information on NORD‐STAR data can be obtained by contacting the corresponding author.
