# Supplementary material for: Flare risk after oral glucocorticoid bridging with methotrexate or intra‐articular bridging with triple therapy in early rheumatoid arthritis
Source: J Intern Med. 2026 Apr 6;299(6):741–53. doi: 10.1111/joim.70094 (PMC13137394; doi:10.1111/joim.70094)
Supplement: Supplementary file 1 — Table S1: Baseline characteristics and medications of patients included in the subgroup analysis (n = 597). Table S2: Baseline characteristics and medications of patients included in the remission subgroup analysis (n = 337). Table S3: Missingness of CDAI and CDAI flare data, stratified by treatment group. Figure S1: Crude subgroup analyses. Figure S2: Disease activity stratified by flare status at Week 40. Figure S3: Disease activity stratified by flare status at Week 40 for the remission subgroup. Figure S4: Assessment of the influence of Week 12 CDAI flare on disease activity up to Week 48. [file JOIM-299-741-s001.docx]

| Supplementary Table 1. Baseline characteristics and medications of patients included in the subgroup analysis (n=597). | | | | | | |
| --- | --- | --- | --- | --- | --- | --- |
| Baseline characteristics | **bDMARD group** | | **Injection GC group** | | **Oral GC group** | |
| n (patients) | 487 |  | 59 |  | 51 | |
| Treatment added to MTX | bDMARD | | IA GC injections, SSZ and HCQ | | oral GC | |
| Female (%) | 68 |  | 71 |  | 71 |  |
| Age, years | 53.9 | (14.6) | 53.8 | (14.8) | 53.3 | (16.4) |
| Diagnosis duration, days, median (IQR) | 8 | (1–19) | 13 | (0–28) | 6 | (0–8) |
| Body-mass index, kg/m2 | 26.3 | (5.0) | 26.5 | (6.0) | 25.1 | (3.7) |
| Smoking (%) |  |  |  |  |  |  |
| Current smoker | 23 |  | 17 |  | 14 |  |
| Former smoker | 36 |  | 46 |  | 45 |  |
| Non-smoker | 40 |  | 37 |  | 41 |  |
| ACPA positive (%) | 83 |  | 81 |  | 84 |  |
| RF positive (%) | 75 |  | 72 |  | 75 |  |
| CDAI score | 27.6 | (11.7) | 26.4 | (10.7) | 28.9 | (12.2) |
| DAS28-CRP* | 5.0 | (1.0) | 4.9 | (0.9) | 5.0 | (1.1) |
| Swollen joint count (28 joints) | 7.7 | (5.0) | 7.1 | (4.8) | 9.0 | (5.1) |
| Tender joint count (28 joints) | 9.0 | (5.9) | 9.0 | (5.6) | 10.2 | (5.9) |
| Patient’s global assessment of disease activity (mm) | 58 | (23) | 59 | (24) | 48 | (22) |
| Physician’s global assessment of disease activity (mm) | 50 | (18) | 45 | (20) | 49 | (19) |
| C-reactive protein, mg/L, median (IQR) | 11 | (4–23) | 11 | (4–23) | 9 | (4–23) |
| Medications of interest |  |  |  |  |  |  |
| Cumulative number of IA GC injections before week 16, median (IQR) | 1 | (0–3) | 5 | (4–8) | 0 | (0–0) |
| Cumulative number of IA GC injections between 16 and 48 weeks, median (IQR) | 0 | (0–0) | 0 | (0–1) | 0 | (0–0) |
| MTX dose at 24 weeks (mg) | 20.5 | (6.1) | 22.1 | (5.0) | 22.1 | (4.1) |
| MTX dose at 48 weeks (mg) | 19.3 | (6.8) | 22.2 | (5.0) | 21.0 | (5.2) |

Data are mean (SD), unless otherwise specified. ACPA=anti-citrullinated protein antibodies. bDMARD refers to a treatment with one of the biologics (certolizumab pegol, abatacept, or tocilizumab). CDAI=Clinical Disease Activity Index. DAS28-CRP=Disease Activity Score of 28 joints, based on C-reactive protein. GC=glucocorticoids. HCQ= hydroxychloroquine. IA=intra-articular. IQR=interquartile range. MTX=methotrexate. RF=rheumatoid factor. SSZ=sulfasalazine.

| Supplementary Table 2. Baseline characteristics and medications of patients included in the remission subgroup analysis (n=337). | | | | | | |
| --- | --- | --- | --- | --- | --- | --- |
| Baseline characteristics | **bDMARD group** | | **Injection GC group** | | **Oral GC group** | |
| n (patients) | 266 |  | 40 |  | 31 | |
| Treatment added to MTX | bDMARD | | IA GC injections, SSZ and HCQ | | oral GC | |
| Female (%) | 64 |  | 70 |  | 58 |  |
| Age, years | 53.0 | (15.0) | 54.8 | (15.1) | 55.5 | (17.6) |
| Diagnosis duration, days, median (IQR) | 8 | (2–19) | 9 | (0–27) | 7 | (1–8) |
| Body-mass index, kg/m2 | 25.3 | (4.1) | 26.6 | (5.6) | 25.1 | (3.8) |
| Smoking (%) |  |  |  |  |  |  |
| Current smoker | 20 |  | 13 |  | 13 |  |
| Former smoker | 37 |  | 53 |  | 48 |  |
| Non-smoker | 43 |  | 35 |  | 39 |  |
| ACPA positive (%) | 84 |  | 88 |  | 77 |  |
| RF positive (%) | 77 |  | 74 |  | 65 |  |
| CDAI score | 25.5 | (10.7) | 24.8 | (9.1) | 26.5 | (11.6) |
| DAS28-CRP* | 4.8 | (1.0) | 4.9 | (0.9) | 4.8 | (1.0) |
| Swollen joint count (28 joints) | 7.5 | (4.8) | 6.7 | (4.1) | 8.4 | (5.1) |
| Tender joint count (28 joints) | 7.9 | (5.4) | 8.3 | (5.2) | 9.2 | (5.7) |
| Patient’s global assessment of disease activity (mm) | 55 | (23) | 57 | (23) | 46 | (22) |
| Physician’s global assessment of disease activity (mm) | 47 | (19) | 41 | (17) | 46 | (18) |
| C-reactive protein, mg/L, median (IQR) | 12 | (4–25) | 11 | (5–22) | 9 | (4–21) |
| Medications of interest |  |  |  |  |  |  |
| Cumulative number of IA GC injections before week 16, median (IQR) | 1 | (0–3) | 5 | (4–8) | 0 | (0–0) |
| Cumulative number of IA GC injections between 16 and 48 weeks, median (IQR) | 0 | (0–0) | 0 | (0–1) | 0 | (0–0) |
| MTX dose at 24 weeks (mg) | 21.0 | (5.9) | 22.2 | (4.4) | 21.5 | (4.6) |
| MTX dose at 48 weeks (mg) | 19.6 | (6.8) | 21.7 | (5.4) | 20.6 | (6.0) |

Data are mean (SD), unless otherwise specified. ACPA=anti-citrullinated protein antibodies. bDMARD refers to a treatment with one of the biologics (certolizumab pegol, abatacept, or tocilizumab). CDAI=Clinical Disease Activity Index. DAS28-CRP=Disease Activity Score of 28 joints, based on C-reactive protein. GC=glucocorticoids. HCQ= hydroxychloroquine. IA=intra-articular. IQR=interquartile range. MTX=methotrexate. RF=rheumatoid factor. SSZ=sulfasalazine.

| Supplementary Table 3. Missingness of CDAI and CDAI flare data, stratified by treatment group. | | | |
| --- | --- | --- | --- |
| Baseline characteristics | **bDMARD group (n=595)**  Missing data, n (%) | **Injection GC group (n=80)**  Missing data, n (%) | **Oral GC group (n=135)**  Missing data, n (%) |
| CDAI |  |  |  |
| CDAI at baseline | 5 (0.8) | 0 (0) | 1 (0.7) |
| CDAI at week 4 | 36 (6.1) | 2 (2.5) | 7 (5.2) |
| CDAI at week 12 | 36 (6.1) | 7 (8.8) | 6 (4.4) |
| CDAI at week 24 | 57 (9.6) | 8 (10.0) | 15 (11.1) |
| CDAI at week 32 | 88 (14.8) | 18 (22.5) | 22 (16.3) |
| CDAI at week 40 | 96 (16.1) | 20 (25.0) | 29 (21.5) |
| CDAI at week 48 | 96 (16.1) | 10 (12.5) | 32 (23.7) |
| CDAI flare |  |  |  |
| CDAI flare at week 12 | 58 (9.7) | 8 (10.0) | 12 (8.9) |
| CDAI flare at week 24 | 64 (10.8) | 11 (13.8) | 16 (11.9) |
| CDAI flare at week 32 | 89 (15.0) | 19 (23.8) | 23 (17.0) |
| CDAI flare at week 40 | 108 (18.2) | 21 (26.3) | 30 (22.2) |
| CDAI flare at week 48 | 114 (19.2) | 20 (25.0) | 35 (25.9) |

CDAI=Clinical Disease Activity Index. CDAI flare was defined as an increase in the CDAI score of ≥4.5.

**Supplementary Figure 1.** Crude subgroup analyses.


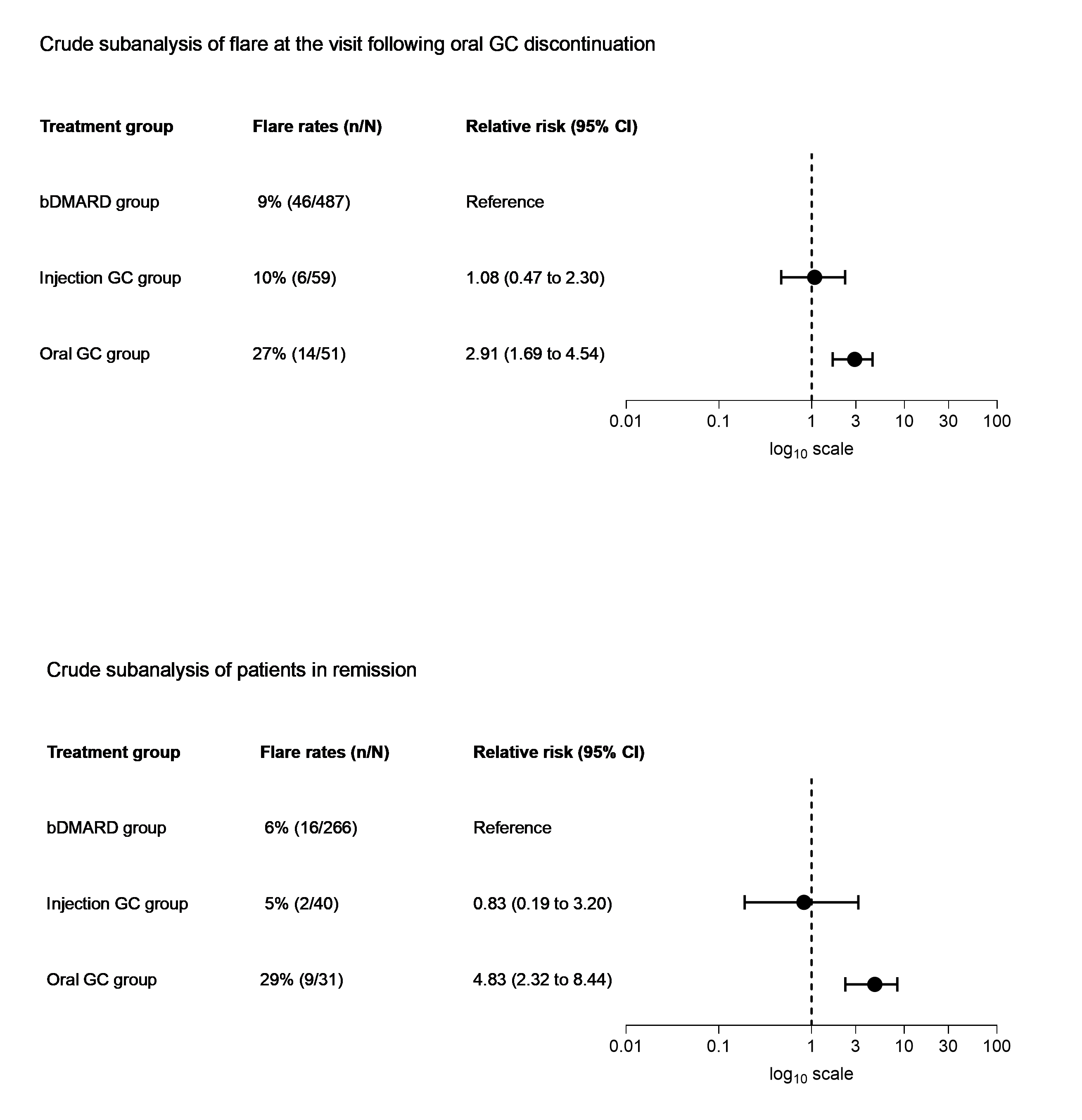


Crude relative risk of CDAI flare at the visit following oral GC discontinuation (week 40), among patients who discontinued GC:s between weeks 32 and 40, injection GC group compared with the reference bDMARD group. The second subgroup analysis was restricted to patients in remission; bDMARD refers to a treatment with one of the biologics (certolizumab pegol, abatacept, or tocilizumab). CDAI=Clinical Disease Activity Index. GC=glucocorticoids.


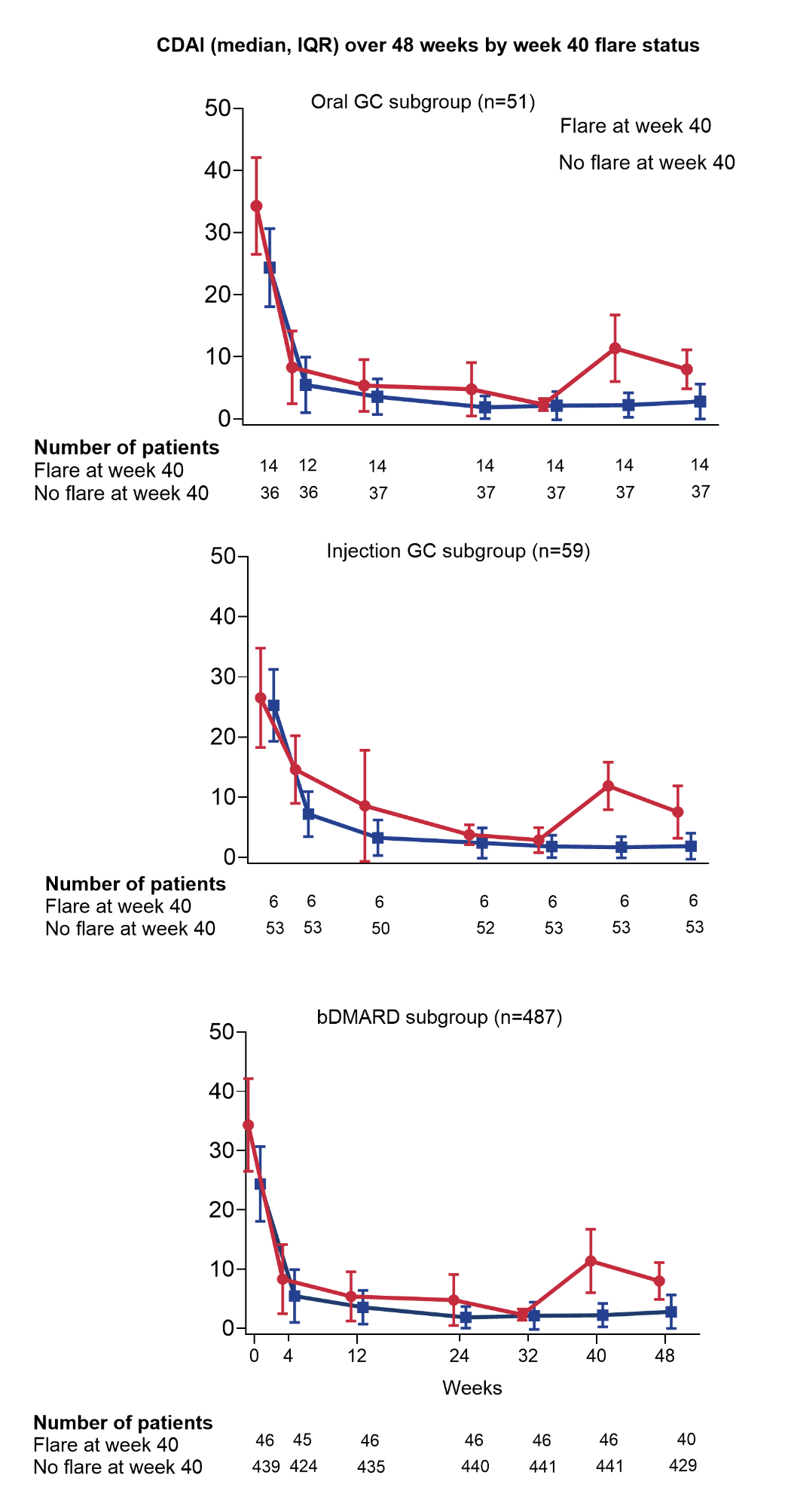
**Supplementary Figure 2.** Disease activity stratified by flare status at week 40.

**Supplementary Figure 3.** Disease activity stratified by flare status at week 40 for the remission subgroup.


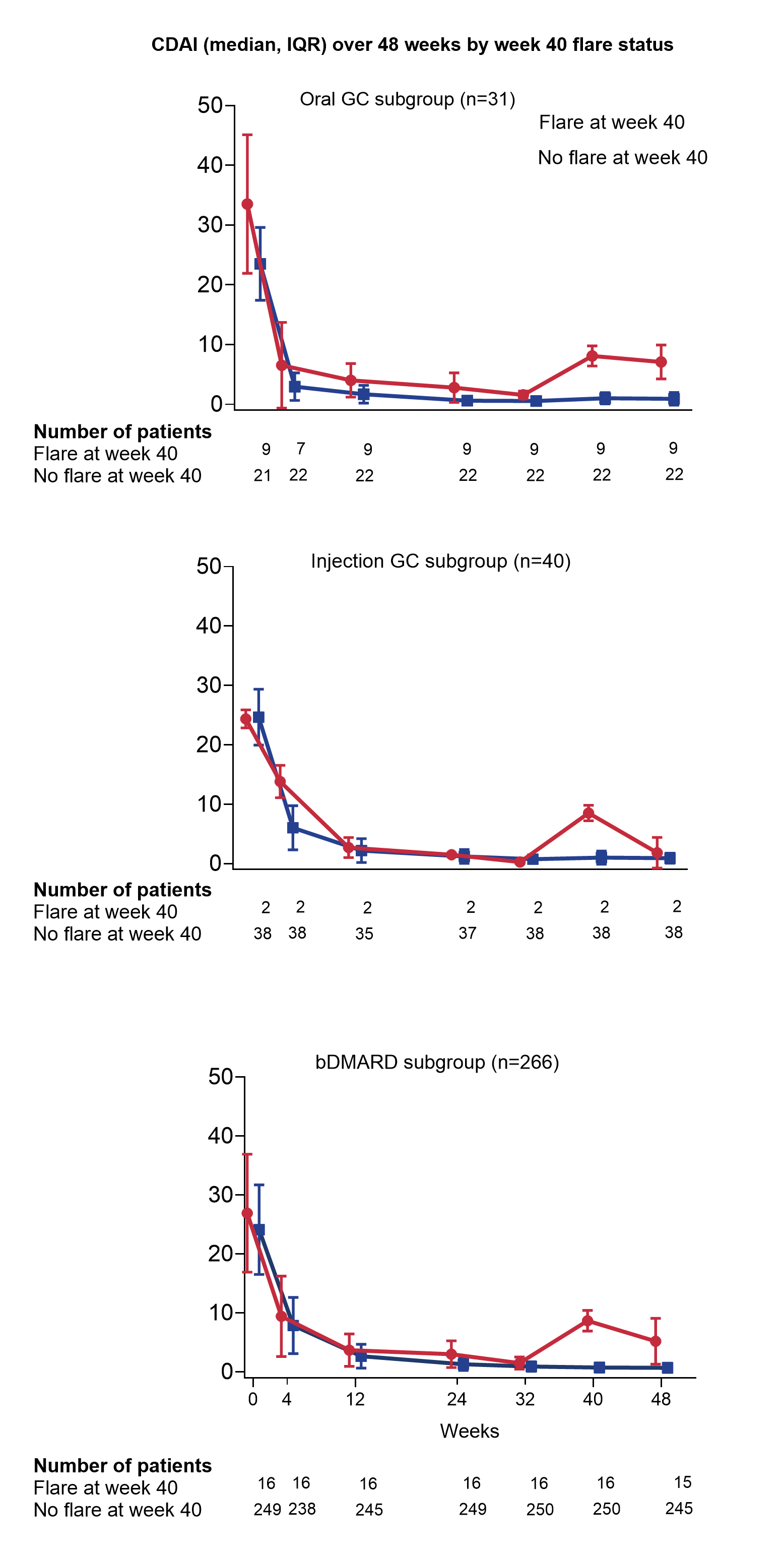


**
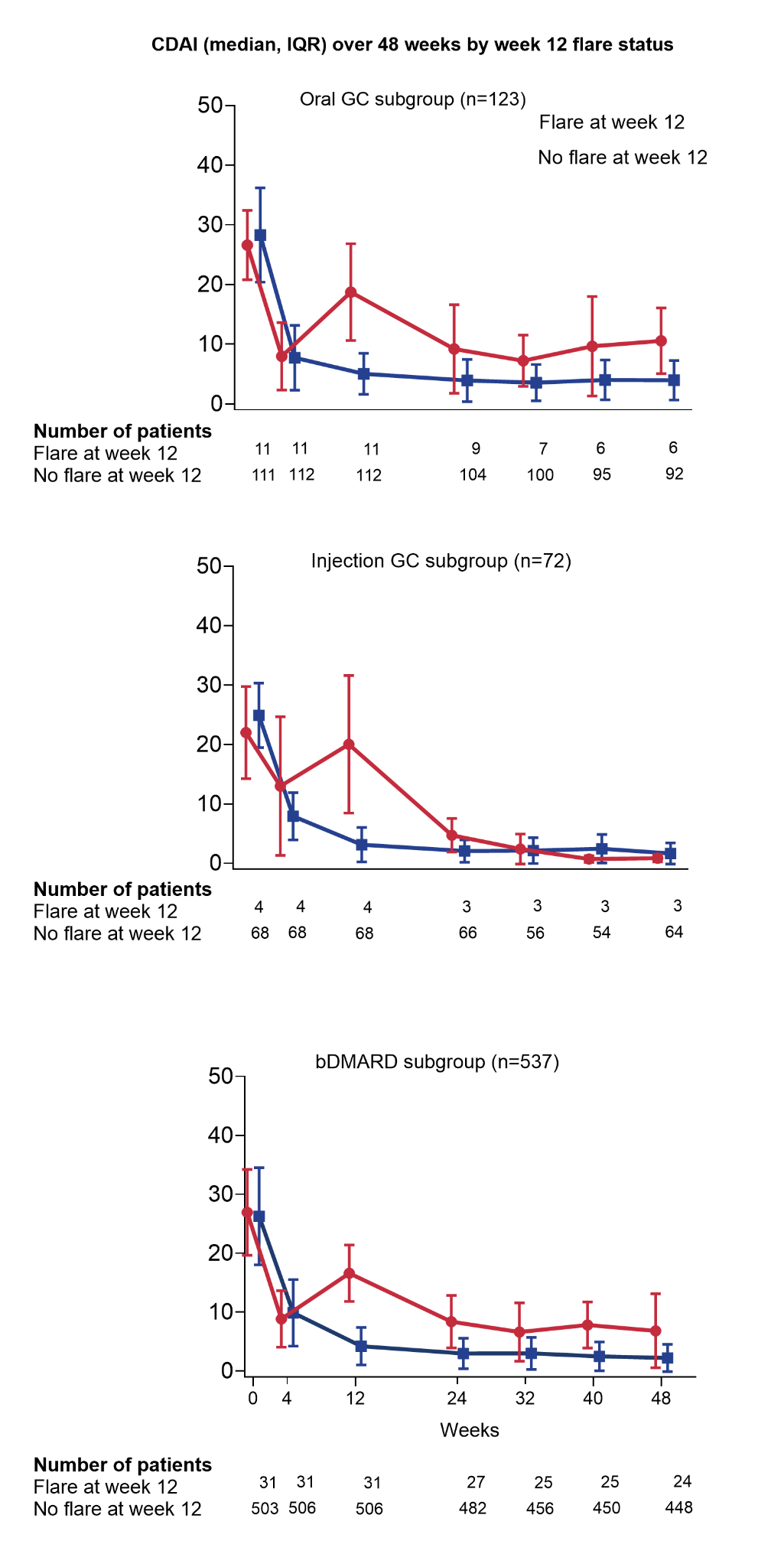
Supplementary Figure 4.** Assessment of the influence of week 12 CDAI flare on disease activity up to week 48.
